# ERICA: leisure-time physical inactivity in Brazilian adolescents

**DOI:** 10.1590/S01518-8787.2016050006683

**Published:** 2016-02-02

**Authors:** Felipe Vogt Cureau, Thiago Luiz Nogueira da Silva, Katia Vergetti Bloch, Elizabeth Fujimori, Dilson Rodrigues Belfort, Kênia Mara Baiocchi de Carvalho, Elisa Brosina de Leon, Mauricio Teixeira Leite de Vasconcellos, Ulf Ekelund, Beatriz D Schaan

**Affiliations:** I Programa de Pós-Graduação em Endocrinologia. Universidade Federal do Rio Grande do Sul. Porto Alegre, RS, Brasil; II Department of Sports Medicine. Norwegian School of Sports Science. Oslo, Norway; III Instituto de Estudos em Saúde Coletiva. Universidade Federal do Rio de Janeiro. Rio de Janeiro, RJ, Brasil; IV Departamento de Enfermagem em Saúde Coletiva. Escola de Enfermagem. Universidade de São Paulo. São Paulo, SP, Brasil; V Universidade Federal do Amapá. Macapá, AP, Brasil; VI Departamento de Nutrição. Universidade de Brasília. Brasília, DF, Brasil; VII Faculdade de Educação Física e Fisioterapia. Universidade Federal do Amazonas. Manaus, AM, Brasil; VIII Escola Nacional de Ciências Estatísticas. Fundação Instituto Brasileiro de Geografia e Estatística. Rio de Janeiro, RJ, Brasil; IX Serviço de Endocrinologia. Hospital de Clínicas de Porto Alegre. Universidade Federal do Rio Grande do Sul. Porto Alegre, RS, Brasil

**Keywords:** Adolescent, Motor Activity, Sedentary Lifestyle, Prevalence, Cross-Sectional Studies

## Abstract

**OBJECTIVE:**

To evaluate the prevalence of leisure-time physical inactivity in Brazilian adolescents and their association with geographical and sociodemographic variables.

**METHODS:**

The sample was composed by 74,589 adolescents participating in the Study of Cardiovascular Risks in Adolescents (ERICA). This cross-sectional study of school basis with national scope involved adolescents aged from 12 to 17 years in Brazilian cities with more than 100 thousand inhabitants. The prevalence of leisure-time physical inactivity was categorized according to the volume of weekly practice (< 300; 0 min). The prevalences were estimated for the total sample and by sex. Poisson regression models were used to assess associated factors.

**RESULTS:**

The prevalence of leisure-time physical inactivity was 54.3% (95%CI 53.4-55.2), and higher for the female sex (70.7%, 95%CI 69.5-71.9) compared to the male (38.0%, 95%CI 36.7-39.4). More than a quarter of adolescents (26.5%, 95%CI 25.8-27.3) reported not practicing physical activity in the leisure time, a condition more prevalent for girls (39.8%, 95%CI 38.8-40.9) than boys (13.4%, 95%CI 12.4-14.4). For girls, the variables that were associated with physical inactivity were: reside in the Northeast (RP = 1.13, 95%CI 1.08-1.19), Southeast (RP = 1.16, 95%CI 1.11-1.22) and South (RP = 1.12, 95%CI 1.06-1.18); have 16-17 years (RP = 1.06, 95%CI 1.12-1.15); and belong to the lower economic class (RP = 1.33, 95%CI 1.20-1.48). The same factors, except reside in the Southeast and South, were also associated with not practicing physical activity in the leisure time for the same group. In males, as well as the region, being older (p < 0.001) and declaring to be indigenous (RP = 0.37, 95%CI 0.19-0.73) were also associated with not practicing physical activities in the leisure time.

**CONCLUSIONS:**

The prevalence of leisure-time physical inactivity in Brazilian adolescents is high. It presents regional variations and is associated with age and low socioeconomic status. Special attention should be given to girls and to those who do not engage in any physical activity during the leisure time, so that they can adopt a more active lifestyle.

## INTRODUCTION

Physical inactivity is one of the main risk factors for the development of chronic diseases^25^. According to the study by Lee et al.^17^, it is possible to assign to physical inactivity the occurrence of 5.3 million deaths in the world only in the year of 2008. In Brazil, 13.0% of deaths in 2008 were attributed to physical inactivity^17^. This information relate directly to the adult population, but also to the population of adolescents, since active adolescents have a higher chance to remain active in adulthood^1^ ^,^ ^22^. Practicing physical activities in this age group is associated with immediate benefits, such as prevention of cardiovascular and metabolic risk factors^11^ ^,^ ^18^, and also predicts better health in adulthood^15^.

Nevertheless, the world prevalence of physical inactivity in adolescents (13-15 years) is of 80.0%, considering the recommendation of at least 60 min/day of moderate or vigorous physical activity^14^. In Brazil, a systematic review with meta-analysis showed great variation in the prevalence of physical inactivity, both in male adolescents (2.0%-80.0%) and females (14.0%-91.0%). The study also noted the data shortage in the North (one study) and Midwest (no studies) regions, and the great variability in the definition of physical inactivity^2^.

The *Pesquisa Nacional de Saúde do Escolar* (PeNSE – National Survey of Students’ Health), which involved Brazilian adolescents of the ninth year of basic education to all regions of the Country and used the cutting point of less than 300 min/week to define physical inactivity, found prevalence of 57.0% in 2009^13^. The same survey conducted in 2012, with some changes in the methodology and in the research tool, found prevalence of physical inactivity of 71.0%, being greater in the Northeast (76.0%) and lower in the South region (65.0%)^19^.

These data reinforce the need for continuous monitoring of the population levels of physical activity in adolescence, to provide more effective intervention strategies. Thus, the objective of this study was to evaluate the prevalence of leisure-time physical inactivity in Brazilian adolescents and their association with geographical and sociodemographic variables.

## METHODS

The sample was composed of adolescents participating in the Study of Cardiovascular Risks in Adolescents (ERICA). This is a cross-sectional study of school basis with national scope, including public and private schools located in urban and rural areas. The data collection took place between February 2013 and November 2014. We assessed 85,615 students, residents of Brazilian municipalities with more than 100 thousand inhabitants (medium and large municipalities). In this study, we used data from 74,589 adolescents aged between 12 and 17 years, who have fully completed the block on practice of physical activity in ERICA.

For sampling, the target population of ERICA was divided into 32 geographical strata: 26 state capitals and the Federal District, and five more sets representing other municipalities with more than 100 thousand inhabitants in each macro-region of the country. The sample size was calculated for each stratum, to ensure representative estimates of each one. The schools were selected with probability proportional to a size directly proportional to the number of students at the school in the school years considered, and inversely proportional to the distance between the municipality and the capital of the state in question. In total, 1,247 schools participated in this study, of 124 municipalities with more than 100 thousand inhabitants.

In the second stage, we selected three combinations of shift (morning and afternoon) and eligible years (seventh, eighth and ninth years of Elementary School and first, second and third years of High School). In the third stage, a class of each combination of shift and year was selected by means of equiprobability. All students of the selected classes were invited to participate of ERICA. More details about the design of the sample of ERICA can be obtained in a previous publication^23^.

The research protocol of ERICA was described by Bloch et al.^4^ In summary, after being selected, the schools were contacted and invited to participate in the study. ERICA data collection involved the application of a structured questionnaire, anthropometric assessment, measurement of blood pressure and blood collection. The variables used in this study were obtained by structured questionnaire, filled by the adolescents on their own, inserted into an electronic data collector (personal digital assistant – PDA).

To determine the level of physical activity of adolescents, we used an adaptation of the Self-Administered Physical Activity Checklist^20^, which consists of a list of 24 modes and allows the adolescents to report frequency (days) and the time (hours and minutes) that they performed, in the last week, some of the activities listed. This questionnaire has already been used in other research on Brazil^3^ ^,^ ^7^ and the version used in ERICA was validated in Brazilian adolescents^12^. For estimates of the prevalence of leisure-time physical inactivity, were used only questions relating to this domain (21 questions).

To determine the level of physical activity, we calculated the product between time and frequency in each activity and the sum of the times obtained. The adolescents who not accumulated at least 300 min/week of physical activity in the leisure time were considered inactive^24^. The prevalence of adolescents who did not report any practice of physical activity during the leisure time in the week preceding the survey (0 min/week) was also evaluated.

Among the independent variables, we analyzed the geographic location according to the region of residence of adolescents (North, Northeast, Midwest, Southeast and South) and the following sociodemographic characteristics: sex; age in full years, later recategorized (12-13, 14-15, 16-17 years); and self-declared skin color (white, black, brown (mixed), yellow (Asian) or indigenous). Socioeconomic status was defined using the Critério Brasil^5^, which considers possession of goods, presence of housekeeper and education of the chief of the household. The score obtained can range from 0 to 46 points. This score was categorized into levels according to recommendation of the instrument: A (35-46 points), B (23-34 points), C (14-22 points), D (8-13 points) and E (0-7 points). Classes D and E were regrouped in the same category, due to low frequency.

The analysis of data involved the estimation of prevalence of leisure-time physical inactivity (< 300 min/week) and of those who did not perform any physical activity during the leisure time at all (0 min/week), with their respective 95% confidence intervals (95%CI). The prevalences were estimated for the entire sample, stratified by sex and described according to the independent variables studied.

The associations of the two categories of physical inactivity with exposure variables were investigated using Poisson regression. The adjusted model was built with only one level of the input variables, which were taken from those with less influence to obtain the final model. We analyzed the presence of multicollinearity. The adjustment of the model was evaluated by goodness-of-fit test. The presence of interactions was tested by multiplicative approach through heterogeneity test. Poisson regression models showed good overall adjustment and we did not observe multicollinearity problems or significant interactions.

The results of the sample were expanded to represent the Brazilian population of schoolchildren in Brazilian cities with more than 100 thousand inhabitants. The sample weights calculated for the study were considered to obtain the estimates^23^. Analyses were conducted in Stata 14 with a significance level of 5%.

All adolescents agreed in writing to participate in the study; five states also requested signing of an informed consent signed by the parent or legal guardian, according to the determination of the Research Ethics Committees (REC) or State Secretariat of Education. ERICA was approved by the REC of each of the 27 Federation units in Brazil.

## RESULTS

Half of the sample was composed of adolescents who studied in strata of the Southeast and Northeast regions, 75.0% in capitals, and 79.0% in public schools. The vast majority of the schools were located in urban areas (98.0%). The girls are the majority in the sample (55.0%), as well as those in the age group of 14-15 years (37.0%), brown skin color (52.0%) and intermediate (B and C) economic classes (86.0%).

**Figure 1:**
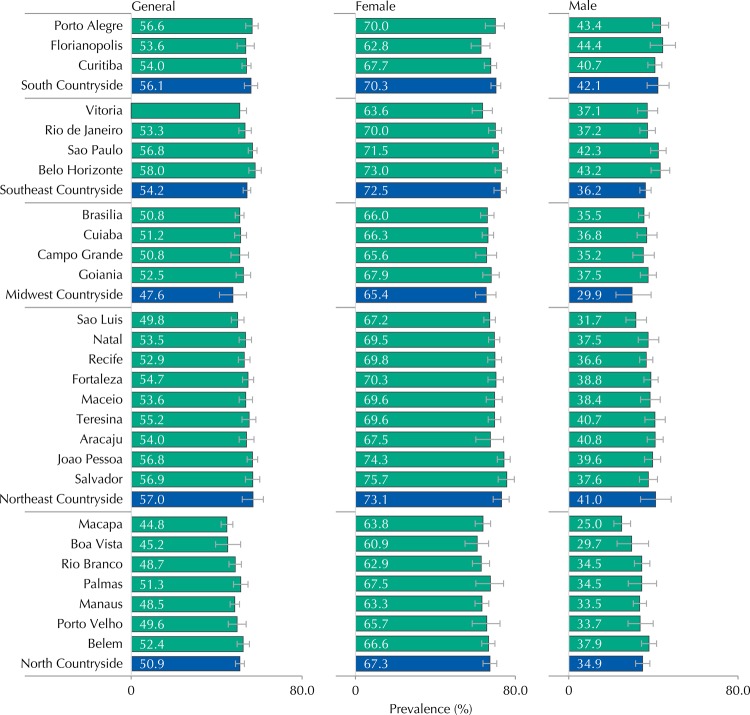
Prevalence of leisure-time physical inactivity (< 300 min/week) in adolescents from the 27 Brazilian capitals (green bars) and set of municipalities with more than 100 thousand inhabitants, not capitals of regions, according to sex. ERICA, 2013-2014.

The prevalence of leisure-time physical inactivity was 54.3% (95%CI 53.4-55.2), higher for the female sex (70.7%, 95%CI 69.5-71.9) compared to the male (38.0%, 95%CI 36.7-39.4) (Table 1). Figure 1 shows the prevalence of adolescents who not accumulated at least 300 min/week of physical activity in the leisure time. The results point to Belo Horizonte (58.0%, 95%CI 55.1-60.9) as the capital with the highest prevalence of general leisure-time physical inactivity, while Macapa (44.8%, 95%CI 42.1-47.6) was the capital with the lowest prevalence. The highest prevalence of physical inactivity by sex were observed in Salvador (75.7%, 95%CI 71.4-79.5) and Florianopolis (44.4%, 95%CI 38.5-50.4) for female and male sexes, respectively (Figure 1).

**Table 1:** Sample size, estimate of the population of adolescent students, prevalence and prevalence ratios of leisure-time physical inactivity and no physical activity according to macro-region and sociodemographic characteristics. ERICA, Brazil, 2013-2014.

Variable	Sample (n)^a^	Population (N)^b^	Physical inactivity (< 300 min/week)				No physical activity (0 min/week)			
	
			%	95%CI	PR_adjusted_^c^	95%CI	%	95%CI	PR_adjusted_^c^	95%CI
Brazil	74,589	10,147,700	54.3	53.4-55.2	-	-	26.5	25.8-27.3	-	-
Macro-region										
North	15,073	855,362	49.8	48.7-50.9	1.00	-	27.2	26.1-28.3	1.00	-
Northeast	23,167	2,165,033	55.7	53.2-58.1	1.14	1.07-1.21	29.5	27.8-31.4	1.13	1.04-1.23
Southeast	17,080	5,153,506	54.8	53.5-56.1	1.17	1.12-1.22	26.5	25.3-27.7	1.04	0.96-1.12
South	9,542	1,195,789	55.8	53.5-58.1	1.16	1.09-1.23	22.9	20.9-25.1	0.88	0.76-1.01
Midwest	9,727	778,010	50.1	47.8-52.4	1.04	0.98-1.10	23.6	22.0-25.2	0.92	0.83-1.00
Sex										
Female	41,225	5,052,137	70.7	69.5-71.9	1.00	-	39.8	38.8-40.9	1.00	-
Male	33,364	5,095,563	38.0	36.7-39.4	0.53	0.51-0.56	13.4	12.4-14.4	0.32	0.29-0.36
Age (years)										
12-13	20,571	3,562,176	52.0	50.6-53.4	1.00	-	21.0	19.8-22.2	1.00	-
14-15	27,889	3,550,879	54.2	52.9-55.6	1.06	1.02-1.11	26.6	25.3-27.8	1.35	1.24-1.46
16-17	26,129	3,034,645	57.1	55.5-58.6	1.11	1.06-1.16	33.1	31.7-34.4	1.66	1.54-1.80
Skin color										
White	26,477	4,099,110	55.4	54.1-56.6	1.00	-	26.3	25.1-27.6	1.00	-
Black	5,654	821,258	47.9	44.5-51.3	0.87	0.80-0.95	25.8	22.8-29.0	0.94	0.80-1.11
Brown	37,984	4,943,938	54.6	53.5-55.7	0.96	0.93-0.99	26.6	25.5-27.7	0.88	0.82-0.95
Yellow	1,881	215,840	54.0	49.1-58.8	0.99	0.90-1.09	26.4	22.9-30.3	0.96	0.83-1.11
Indigenous	561	67,554	39.4	29.8-50.0	0.68	0.56-0.82	15.1	11.0-20.2	0.63	0.44-0.90
Economic class										
A (high)	6,336	1,100,099	42.2	38.5-46.1	1.00	-	15.9	13.4-18.7	1.00	-
B	25,258	5,495,819	51.4	50.0-52.8	1.14	1.04-1.25	23.4	22.3-24.5	1.30	1.09-1.54
C	17,339	3,389,885	58.5	57.0-60.1	1.24	1.13-1.36	30.2	28.9-32.5	1.49	1.25-1.78
D-E (low)	936	161,898	64.6	58.0-70.6	1.33	1.17-1.51	32.7	26.8-39.2	1.49	1.15-1.93

ERICA: Study of Cardiovascular Risks in Adolescents; PR: prevalence ratio

^a^ Non-weighted values.

^b^ Population of adolescents, according to the Brazilian Institute of Geography and Statistics.

^c^ Adjusted model for macro-region, sex, age and economic class.

**Figure 2:**
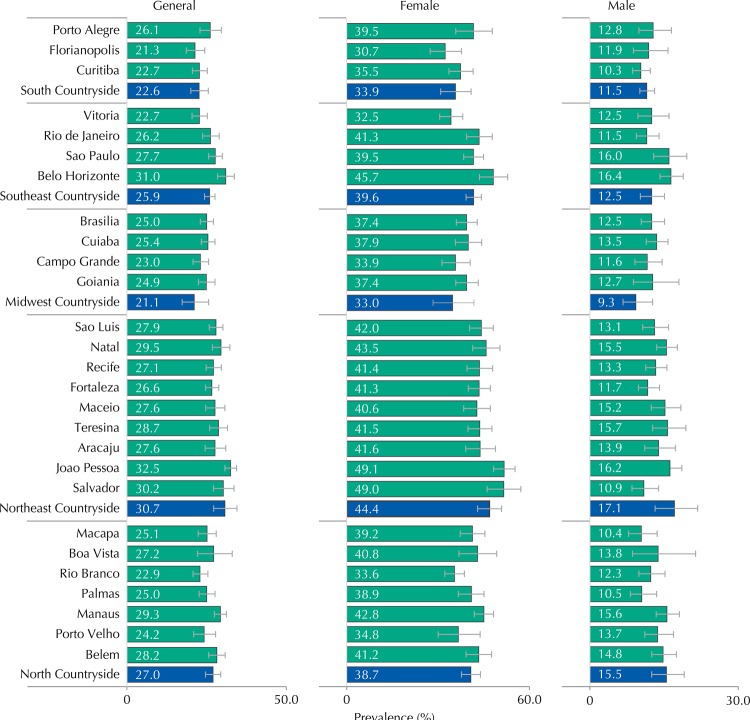
Prevalence (%) of no physical activity during the leisure time (0 min/week) in adolescents of the 27 Brazilian capitals (green bars) and set of municipalities with more than 100,000 inhabitants, not capitals of regions, according to sex. ERICA, 2013-2014.

Among Brazilian adolescents, 26.5% (95%CI 25.8-27.3) reported not to perform physical activity during the leisure time (0 min/week), with higher prevalence in females (39.8%, 95%CI 38.8-40.9%) than males (13.4%, 95%CI 12.4-14.4) (Table 1). In Joao Pessoa, the capital with higher prevalence of no physical activity, one every three adolescents does not practice any physical activity during the leisure time, a ratio that changes to one for every two when considering the female sex (Figure 2).

Table 1 presents the prevalence and adjusted prevalence ratios in the total sample, to physical inactivity and no physical activity according to the independent variables. The highest prevalence of leisure-time physical inactivity were observed in the Northeast for any physical activity; and in the Northeast and South when the cutoff point was at least 300 min/week. In the adjusted model, the prevalence of physical inactivity in both categories is associated directly with age and inversely with economic level (p-value for linear trend < 0.05).

After stratification by sex, the highest prevalence of physical inactivity were in the Northeast and Southeast in the female sex, and in the South region between male adolescents. Among girls, the higher age (p < 0.001) and the lowest socioeconomic class (p < 0.001) were associated with higher prevalence of physical inactivity. In male adolescents, the prevalence of physical inactivity tended to increase as the socioeconomic condition decreased (p = 0.003); to declare oneself as indigenous, black or brown were protection factors regarding physical inactivity in the adjusted model (p < 0.05) (Table 2).

**Table 2:** Prevalence (%) and prevalence ratios for leisure-time physical inactivity (< 300 min/week) according to sex, macro-region and sociodemographic characteristics in adolescents. ERICA, Brazil, 2013-2014.

Variable	Physical inactivity (< 300 min/week)							

	Female				Male			
	
	%	95%CI	PR_adjusted_^a^	95%CI	%	95%CI	PR_adjusted_^b^	95%CI
Macro-region								
North	65.4	63.7-67.1	1.00	-	34.1	32.5-35.8	1.00	-
Northeast	72.0	69.9-74.0	1.13	1.08-1.19	39.3	35.8-42.9	1.15	1.01-1.30
Southeast	72.0	69.8-74.1	1.16	1.11-1.22	37.8	35.8-39.7	1.15	1.05-1.26
South	69.7	67.8-71.6	1.12	1.06-1.18	42.1	38.3-46.0	1.19	1.05-1.34
Midwest	66.1	63.8-68.2	1.05	0.99-1.10	34.1	31.1-37.1	1.02	0.92-1.13
Age (years)								
12-13	66.3	64.5-68.1	1.00	-	38.0	35.4-40.6	1.00	-
14-15	72.5	70.7-74.3	1.10	1.06-1.15	36.1	34.3-38.0	0.99	0.90-1.10
16-17	73.8	71.9-75.6	1.12	1.07-1.18	40.3	38.2-42.4	1.09	0.99-1.20
Skin color								
White	70.9	69.2-72.5	1.00	-	40.4	38.3-42.6	1.00	-
Black	67.4	63.1-71.4	0.96	0.88-1.03	34.4	30.8-38.2	0.75	0.64-0.89
Brown	71.3	69.9-72.6	0.99	0.96-1.03	36.5	34.8-38.2	0.91	0.84-0.98
Yellow	65.5	58.9-71.5	0.94	0.86-1.02	41.7	34.7-49.0	1.08	0.89-1.30
Indigenous	62.0	51.4-71.5	0.86	0.71-1.05	29.3	16.4-46.4	0.52	0.38-0.71
Economic class								
A (high)	59.4	55.1-63.6	1.00	-	31.4	27.0-36.2	1.00	-
B	68.1	66.4-69.8	1.13	1.03-1.23	35.8	33.9-37.8	1.17	1.00-1.37
C	73.5	71.4-75.4	1.22	1.13-1.32	39.9	37.3-42.6	1.33	1.12-1.59
D-E (low)	80.0	73.9-85.0	1.33	1.20-1.48	40.5	29.0-53.1	1.34	0.96-1.87

ERICA: Study of Cardiovascular Risks in Adolescents; PR: prevalence ratio

^a^ Adjusted model for macro-region, age and economic class.

^b^ Adjusted model for macro-region, age, economic class and skin color.

Concerning the non-practice of physical activity (Table 3), we also observed an increasing gradient of the prevalence of no physical activity at leisure with increasing age in both sexes (p < 0.001). Worst socioeconomic condition was associated with higher prevalence of no leisure-time physical activity only among female adolescents (p = 0.001).

**Table 3:** Prevalence (%) and prevalence ratios for no physical activity (0 min/week) in the leisure time according to sex, macro-region and sociodemographic characteristics in adolescents. ERICA, Brazil, 2013-2014.

Variables	No physical activity (0 min/week)							

	Female				Male			
	%	95%CI	PR_adjusted_^a^	95%CI	%	95%CI	PR_adjusted_^b^	95%CI
Macro-region								
North	39.8	38.3-41.3	1.00	-	14.6	13.3-16.1	1.00	-
Northeast	44.0	42.1-46.0	1.17	1.08-1.27	15.0	13.1-17.2	0.99	0.80-1.23
Southeast	39.9	38.2-41.7	1.07	0.98-1.17	13.2	11.5-15.0	0.90	0.72-1.13
South	34.6	31.1-38.2	0.90	0.78-1.06	11.5	10.3-12.7	0.74	0.58-0.93
Midwest	35.6	33.2-38.2	0.96	0.87-1.06	11.5	10.1-13.1	0.77	0.62-0.95
Age (years)								
12-13	31.5	29.6-33.5	1.00	-	10.6	9.3-13.4	1.00	-
14-15	41.1	39.2-43.1	1.32	1.19-1.45	12.2	10.8-13.7	1.47	1.20-1.80
16-17	48.0	46.3-49.6	1.56	1.42-1.72	18.0	16.4-19.8	2.01	1.68-2.41
Skin color								
White	39.3	37.3-41.4	1.00	-	13.8	12.5-15.3	1.00	-
Black	42.5	38.4-46.7	0.97	0.84-1.13	14.2	10.9-18.4	0.87	0.59-1.27
Brown	39.7	38.1-41.3	0.91	0.84-0.98	12.4	11.2-13.8	0.80	0.69-0.93
Yellow	38.4	32.9-44.1	0.96	0.83-1.11	13.7	9.8-18.8	0.96	0.63-1.48
Indigenous	36.0	26.9-46.3	0.80	0.55-1.17	5.5	3.2-9.3	0.37	0.19-0.73
Economic class								
A (high)	25.9	21.2-31.3	1.00	-	9.5	7.2-12.5	1.00	-
B	35.6	33.7-37.5	1.34	1.09-1.65	12.0	10.7-13.5	1.29	0.97-1.70
C	44.2	42.0-46.4	1.60	1.30-1.97	12.7	11.0-14.5	1.32	0.95-1.83
D-E (low)	43.4	34.5-52.7	1.54	1.18-2.00	16.1	10.4-24.2	1.51	0.88-2.59

ERICA: Study of Cardiovascular Risks in Adolescents; RP: prevalence ratio

^a^ Adjusted model for macro-region, age and economic class.

^b^ Adjusted model for macro-region, age, skin color and economic class.

## DISCUSSION

ERICA data showed that more than half of Brazilian adolescents, residents of cities of medium and large size, do not reach the recommendation of at least 300 min/week of physical activity for health promotion. This percentage is even higher among girls, surpassing 70.0%. Equally disturbing is the fact that one in four adolescents do not practice any physical activity in the leisure time, a prevalence that approaches 50.0% among girls in some capitals.

The measurement of population levels of physical activity, especially in childhood and adolescence, is quite complex and to compare studies is difficult. The use of questionnaires does not always produce accurate estimates, which can lead to misclassification, and its agreement with direct measures is only partial^21^. However, using direct measurement methods, such as accelerometer, would be infeasible in a study as ERICA due to high costs and the complex logistics involved. The use of an already validated instrument^12^ increases the reliability of the data, however it does not reduce the difficulty of comparisons due to the different instruments used and the different domains of physical activity assessed in each study. In this study we decided to investigate only physical activity during the leisure time, because it is the most explored domain on the ERICA questionnaire, when compared to the other issues, and because it is the most important area for the implementation of the recommendations of physical activity among Brazilian adolescents^19^.

The scientific literature shows that the practice of physical activity in adolescence is associated with numerous health benefits during adolescence and with equally important reflections on adulthood^11^ ^,^ ^15^ ^,^ ^18^. Although many studies use the recommendation of at least 300 min/week of moderate to vigorous physical activity for health promotion in adolescents, evidence support that smaller volumes can also bring benefits. These variations are supported by multicausal origins of each morbidity, especially chronic ones, as well as by different physiological mechanisms that can relate physical activity to a certain morbidity. A systematic review showed that 30 min/day of physical activity are sufficient to obtain cardiovascular benefits in health among adolescents^16^. However, a randomized clinical trial involving overweight and inactive young people pointed out that 20 min of aerobic physical activity, practiced five times a week, for 13 weeks, already reduces the risk of developing diabetes, general percentage of fat and visceral fat, and also improves physical fitness when compared to the control group that kept usual routine^8^.

Interventions that encourage the transition from total physical inactivity to a state of action, regardless of the frequency of physical activity initially practiced, can promote immediate impacts. This strategy can work complementary to programs aimed at the maintenance and gradual increase of the practice of physical activity among adolescents. The leisure domain is favorable to the development of interventions.

In this context, school seems to be the ideal space to conduct these interventions, due to the large concentration of adolescents, safety, physical space and professionals able to stimulate and supervise these activities. To increase the effective time of physical practice during physical education classes and to make them more attractive should be a goal, as well as reducing the number of layoffs. Other actions such as sedentary time interruptions during class, active intervals proposition and extracurricular activities are strategies that have been studied and could increase the practice of physical activities in schools and have important consequences outside them^6^. However, these actions must be connected to an increased social support for practice and improving access, making it socially egalitarian, especially in countries of socioeconomic conditions so heterogeneous as Brazil.

The interest in the study of physical (in)activity epidemiology in Brazil is growing, with the majority of publications focused on South-Southeast axis and recent growth of publications in the Northeast region. However, studies conducted in the North and Midwest regions of the Country are still rare in this thematic^2^. In ERICA, these regions showed the lowest prevalence of leisure-time physical inactivity, regardless of sex. The latest edition of PeNSE also noted greater performance of leisure time activities in the North region, and related this result to cultural issues and favorable urban features not always found in more urbanized regions such as the Southeast, Northeast and South of the Country^19^. However, further studies are needed in those regions for a better understanding of the associated and promoters factors of the practice of physical activity.

Higher prevalence of physical inactivity in the female sex is often seen, especially during the leisure time^3^ ^,^ ^14^ ^,^ ^19^. The same occurs with increasing age^10^, being even more pronounced in the transition from adolescence to adulthood^1^, a phase that the adolescents studied have yet to experience. Socioeconomic status was associated with physical inactivity among girls, especially in relation to not reaching the recommendation of at least 300 min/week, reinforcing the interpretations that related this variable to the opportunity to practice structured physical activity (purchase of equipment, transportation, payment of fees, among others) and emotional and social support of family (as permission, stimulus, company to practice and easier access to information)^9^.

The first data about physical inactivity of ERICA help size how challenging is this problem in Brazil. These results are useful for directing actions that seek to reduce physical inactivity in Brazilian adolescents. Future analyses of ERICA can expand this knowledge by assessing, for example, the importance of the school structure, seasonality and climatic variations, and the relation of physical inactivity with several cardiovascular risk factors surveyed.

Investment in stimulus policies to physical activity in adolescents should be treated as priority by Government agendas relating to sport, health and education, as well as research funding institutions. Governmental actions as the *Segundo Tempo*, *Mais Educação* and *Atleta na Escola* programs are important steps that should be expanded. The construction of the Bill of Guidelines and Bases of the National Sport System, which aims to make the investment in sport in a government policy to mainstream the practice of sports in the country, may also be favorable.

These resources should assist in changing the current scenario in a comprehensive manner, making school an important partner for the promotion of activities that involve the whole school community. The development of actions aimed at the transition of those who engage in no physical activity to adopt some practice must be a priority, even if these people do not achieve, at first, the recommendations of physical activity for health.
