# Supplementary material for: ERICA: leisure-time physical inactivity in Brazilian adolescents
Source: Rev Saude Publica. 2016 Feb 2;50(Suppl 1):4s. doi: 10.1590/S01518-8787.2016050006683 (PMC4767040; doi:10.1590/S01518-8787.2016050006683)
Supplement: Translation

# Translation: ERICA: inatividade física no lazer em adolescentes brasileiros

**Authors:** Felipe Vogt Cureau, Thiago Luiz Nogueira da Silva, Katia Vergetti Bloch, Elizabeth Fujimori, Dilson Rodrigues Belfort, Kênia Mara Baiocchi de Carvalho, Elisa Brosina de Leon, Mauricio Teixeira Leite de Vasconcellos, Ulf Ekelund, Beatriz D Schaan

**Affiliations:** I Programa de Pós-Graduação em Endocrinologia. Universidade Federal do Rio Grande do Sul. Porto Alegre, RS, Brasil; II Department of Sports Medicine. Norwegian School of Sports Science. Oslo, Norway; III Instituto de Estudos em Saúde Coletiva. Universidade Federal do Rio de Janeiro. Rio de Janeiro, RJ, Brasil; IV Departamento de Enfermagem em Saúde Coletiva. Escola de Enfermagem. Universidade de São Paulo. São Paulo, SP, Brasil; V Universidade Federal do Amapá. Macapá, AP, Brasil; VI Departamento de Nutrição. Universidade de Brasília. Brasília, DF, Brasil; VII Faculdade de Educação Física e Fisioterapia. Universidade Federal do Amazonas. Manaus, AM, Brasil; VIII Escola Nacional de Ciências Estatísticas. Fundação Instituto Brasileiro de Geografia e Estatística. Rio de Janeiro, RJ, Brasil; IX Serviço de Endocrinologia. Hospital de Clínicas de Porto Alegre. Universidade Federal do Rio Grande do Sul. Porto Alegre, RS, Brasil

**Keywords:** Adolescente, Atividade Motora, Estilo de Vida Sedentário, Prevalência, Estudo Transversal

## Abstract

**OBJETIVO:**

Avaliar a prevalência de inatividade física no lazer em adolescentes brasileiros e sua associação com variáveis geográficas e sociodemográficas.

**MÉTODOS:**

A amostra foi composta por 74.589 adolescentes participantes do Estudo de Riscos Cardiovasculares em Adolescentes (ERICA). Esse estudo transversal de base escolar com abrangência nacional envolveu adolescentes brasileiros de 12 a 17 anos de municípios com mais de 100 mil habitantes. A prevalência de inatividade física no lazer foi categorizada de acordo com o volume de prática semanal (< 300; zero min). As prevalências foram estimadas para o total da amostra analisada e por sexo. Modelos de regressão de Poisson foram utilizados para avaliar fatores associados.

**RESULTADOS:**

A prevalência de inatividade física no lazer foi de 54,3% (IC95% 53,4-55,2), superior no sexo feminino (70,7%, IC95% 69,5-71,9) comparado ao masculino (38,0%, IC95% 36,7-39,4). Mais de um quarto dos adolescentes (26,5%, IC95% 25,8-27,3) referiram não praticar atividade física no lazer, condição mais prevalente no sexo feminino (39,8%, IC95% 38,8-40,9) que no masculino (13,4%, IC95% 12,4-14,4). Para o sexo feminino, as variáveis que se associaram à inatividade física foram: residir nas regiões Nordeste (RP = 1,13, IC95% 1,08-1,19), Sudeste (RP = 1,16, IC95% 1,11-1,22) e Sul (RP = 1,12, IC95% 1,06-1,18); ter 16-17 anos (RP = 1,12, IC95% 1,06-1,15); e pertencer à classe econômica mais baixa (RP = 1,33, IC95% 1,20-1,48). Os mesmos fatores, exceto residir no Sudeste e Sul, também associaram-se com não praticar atividade física no lazer no mesmo grupo. No sexo masculino, além da região, ser mais velho (p < 0,001) e declarar-se indígena (RP = 0,37, IC95% 0,19-0,73) associaram-se à prevalência de não praticar atividade física no lazer.

**CONCLUSÕES:**

A prevalência de inatividade física no lazer em adolescentes brasileiros é elevada, apresenta variações regionais e está associada à idade e ao baixo nível socioeconômico. Especial atenção deve ser dada às meninas e aos que não praticam nenhuma atividade física no lazer, a fim de que estes possam adotar estilo de vida mais ativo.

## INTRODUÇÃO

A inatividade física é um dos principais fatores de risco para o desenvolvimento de doenças crônicas^25^. De acordo com o estudo de Lee et al.^17^, pode-se atribuir à inatividade física a ocorrência de 5,3 milhões de mortes no mundo apenas no ano de 2008. No Brasil, 13,0% das mortes em 2008 foram atribuídas à inatividade física^17^. Essas informações referem-se diretamente à população adulta, porém também relacionam-se à população de adolescentes, já que adolescentes ativos apresentam maior chance de se manterem ativos na idade adulta^1^ ^,^ ^22^. A prática de atividade física nessa faixa etária está associada a benefícios imediatos, como prevenção de fatores de risco cardiovasculares e metabólicos^11^ ^,^ ^18^, bem como prediz melhores condições de saúde na idade adulta^15^.

Apesar disso, a prevalência mundial de inatividade física em adolescentes (13-15 anos) é de 80,0%, considerando-se recomendação de pelo menos 60 min/dia de atividade física moderada ou vigorosa^14^. No Brasil, revisão sistemática com metanálise mostrou grande variação da prevalência de inatividade física, tanto em adolescentes do sexo masculino (2,0%-80,0%), quanto do feminino (14,0%-91,0%). O estudo observou ainda a escassez de dados nas regiões Norte (um estudo) e Centro-Oeste (nenhum estudo) do País e grande variabilidade na definição de inatividade física^2^.

A Pesquisa Nacional de Saúde do Escolar (PeNSE), que envolveu adolescentes brasileiros do nono ano do ensino fundamental de todas as regiões do País e utilizou o ponto de corte de menos que 300 min/semana para definir inatividade física, encontrou prevalência de 57,0% na edição de 2009^13^. O mesmo inquérito realizado em 2012, com algumas alterações metodológicas e no instrumento de pesquisa, encontrou prevalência de inatividade física de 71,0%, sendo maior na região Nordeste (76,0%) e menor na região Sul (65,0%)^19^.

Esses dados reforçam a necessidade de monitoramento contínuo dos níveis populacionais de atividade física na adolescência, a fim de subsidiar estratégias de intervenção mais efetivas. Desta forma, o objetivo deste estudo foi avaliar a prevalência de inatividade física no lazer em adolescentes brasileiros e sua associação com variáveis geográficas e sociodemográficas.

## MÉTODOS

A amostra foi composta por adolescentes participantes do Estudo de Riscos Cardiovasculares em Adolescentes (ERICA), estudo transversal de base escolar com abrangência nacional, incluindo escolas públicas e privadas localizadas em zonas urbanas e rurais. A coleta de dados do estudo ocorreu entre fevereiro de 2013 e novembro de 2014. Foram avaliados 85.615 estudantes moradores de municípios brasileiros com mais de 100 mil habitantes (municípios de médio e grande porte). No presente estudo, foram utilizados dados de 74.589 adolescentes com idades entre 12 e 17 anos que completaram integralmente o bloco relativo à prática de atividade física no ERICA.

Para a amostragem, a população alvo do ERICA foi dividida em 32 estratos geográficos: 26 capitais e o Distrito Federal, e mais cinco conjuntos representando os demais municípios com mais de 100 mil habitantes em cada uma das macrorregiões do País. O tamanho da amostra foi calculado para cada estrato, visando a assegurar estimativas representativas de cada um. As escolas foram selecionadas com probabilidade proporcional a um tamanho diretamente proporcional ao número de alunos da escola nos anos letivos considerados e inversamente proporcional à distância entre o município da escola e a capital da unidade da federação. No total, participaram do estudo 1.247 escolas, em 124 municípios com mais de 100 mil habitantes.

No segundo estágio, foram selecionadas três combinações de turno (manhã e tarde) e anos elegíveis (sétimo, oitavo e nono do ensino fundamental e primeiro, segundo e terceiro do ensino médio). No terceiro estágio, uma turma de cada combinação de turno e ano foi selecionada com equiprobabilidade. Todos os alunos das turmas selecionadas foram convidados a participar do ERICA. Mais detalhes sobre o desenho da amostra do ERICA podem ser obtidos em publicação anterior^23^.

O protocolo de pesquisa do ERICA foi descrito por Bloch et al.^4^ Em resumo, após selecionadas, as escolas foram contatadas e convidadas a participar do estudo. A coleta de dados do ERICA envolveu a aplicação de um questionário estruturado, avaliação antropométrica, aferição de pressão arterial e coleta de sangue. As variáveis utilizadas neste estudo foram obtidas por questionário estruturado, autopreenchido pelo adolescente, inserido em um coletor eletrônico de dados (*personal digital assistant* – PDA).

Para determinação do nível de atividade física dos adolescentes, foi utilizada uma adaptação do *Self-Administered Physical Activity Checklist* ^20^, o qual consiste em uma lista de 24 modalidades e permite que o adolescente informe a frequência (dias) e o tempo (horas e minutos) que praticou, na última semana, alguma das atividades listadas. Este questionário já foi utilizado em outras pesquisas no Brasil^3^ ^,^ ^7^ e a versão utilizada no ERICA foi validada em adolescentes brasileiros^12^. Para estimativas da prevalência de inatividade física no lazer, foram utilizadas apenas questões relativas a este domínio (21 questões).

Para determinação do nível de atividade física, foi calculado o produto entre o tempo e a frequência em cada atividade e calculado o somatório dos tempos obtidos. Os adolescentes que não acumularam pelo menos 300 min/semana de atividade física foram considerados inativos no lazer^24^. A prevalência de adolescentes que não referiram qualquer prática de atividade física no lazer na semana anterior à pesquisa (zero min/semana) também foi avaliada.

Entre as variáveis independentes, foram analisadas a localização geográfica de acordo com a região de residência dos adolescentes (Norte, Nordeste, Centro-Oeste, Sudeste e Sul) e as seguintes características sociodemográficas: sexo, idade em anos completos e posteriormente recategorizada (12-13, 14-15, 16-17 anos), e cor da pele declarada (branca, preta, parda, amarela ou indígena). O nível socioeconômico foi definido pela utilização do Critério Brasil^5^, que considera posse de bens, presença de empregada doméstica e escolaridade do chefe da família. O escore obtido pode variar de zero a 46 pontos. Este escore foi categorizado em níveis conforme recomendação do instrumento: A (35-46 pontos), B (23-34 pontos), C (14-22 pontos), D (8-13 pontos) e E (0-7 pontos). As classes D e E foram reagrupadas em uma mesma categoria, devido a sua baixa frequência.

A análise dos dados envolveu a estimativa da prevalência de inatividade física no lazer (< 300 min/semana) e daqueles que não fizeram qualquer atividade física de lazer (zero min/semana) e respectivos intervalos de confiança de 95% (IC95%). As prevalências foram estimadas para toda a amostra, estratificadas por sexo e descritas segundo as variáveis independentes estudadas.

As associações das duas categorias de inatividade física com variáveis de exposição foram investigadas por meio de regressão de Poisson. O modelo ajustado foi construído com apenas um nível de entrada das variáveis, sendo retiradas aquelas com menor influência até a obtenção do modelo final. Foi analisada a presença de multicolinearidade e o ajuste do modelo foi avaliado pelo teste de *goodness-of-fit*. A presença de interações foi testada por abordagem multiplicativa por meio do teste de heterogeneidade. Os modelos de regressão de Poisson apresentaram bom ajuste global e não foram observados problemas de multicolinearidade ou interações significativas.

Os resultados da amostra foram expandidos para representar a população brasileira de escolares em cidades brasileiras com mais de 100 mil habitantes. Os pesos amostrais calculados para o estudo foram considerados para obtenção das estimativas^23^. As análises foram conduzidas no Stata 14 com nível de significância de 5%.

Todos os adolescentes assentiram por escrito em participar do estudo; em cinco estados, foi solicitado adicionalmente termo de consentimento livre e esclarecido assinado pelos pais ou responsáveis por determinação dos Comitês de Ética em Pesquisa (CEP) ou Secretaria Estadual de Educação. O ERICA foi aprovado pelo CEP de cada uma das 27 unidades da federação.

## RESULTADOS

Metade da amostra foi composta por adolescentes que estudavam nos estratos das regiões Sudeste e Nordeste, 75,0% nas capitais, 79,0% em escolas públicas e a grande maioria em escolas situadas em área urbana (98,0%). As meninas são maioria na amostra (55,0%), assim como aqueles na faixa etária de 14-15 anos (37,0%), de cor da pele parda (52,0%) e das classes econômicas intermediárias B e C (86,0%).

A prevalência de inatividade física no lazer foi de 54,3% (IC95% 53,4-55,2), maior no sexo feminino (70,7%, IC95% 69,5-71,9) que entre adolescentes do sexo masculino (38,0%, IC95% 36,7-39,4) (Tabela 1). A Figura 1 apresenta a prevalência de adolescentes brasileiros que não acumularam pelos menos 300 min/semana de atividade física no lazer. Os resultados apontam Belo Horizonte (58,0%, IC95% 55,1-60,9) como a capital com a maior prevalência geral de inatividade física no lazer, enquanto Macapá (44,8%, IC95% 42,1-47,6) foi a capital com a menor prevalência. As maiores prevalências de inatividade física por sexo foram observadas em Salvador (75,7%, IC95% 71,4-79,5) e Florianópolis (44,4%, IC95% 38,5-50,4) para o sexo feminino e masculino, respectivamente (Figura 1).

**Tabela 1:** Tamanho da amostra, estimativa da população de estudantes adolescentes, prevalências e razões de prevalência para inatividade física e nenhuma atividade física no lazer segundo macrorregião e características sociodemográficas. ERICA, Brasil 2013-2014.

Variável	Amostra (n)^a^	População (N)^b^	Inatividade física (< 300 min/sem)				Nenhuma atividade física (0 min/sem)			
	
			%	IC95%	RP_ajustada_^c^	IC95%	%	IC95%	RP_ajustada_^c^	IC95%
Brasil	74.589	10.147.700	54,3	53,4-55,2	-	-	26,5	25,8-27,3	-	-
Macrorregião										
Norte	15.073	855.362	49,8	48,7-50,9	1,00	-	27,2	26,1-28,3	1,00	-
Nordeste	23.167	2.165.033	55,7	53,2-58,1	1,14	1,07-1,21	29,5	27,8-31,4	1,13	1,04-1,23
Sudeste	17.080	5.153.506	54,8	53,5-56,1	1,17	1,12-1,22	26,5	25,3-27,7	1,04	0,96-1,12
Sul	9.542	1.195.789	55,8	53,5-58,1	1,16	1,09-1,23	22,9	20,9-25,1	0,88	0,76-1,01
Centro-Oeste	9.727	778.010	50,1	47,8-52,4	1,04	0,98-1,10	23,6	22,0-25,2	0,92	0,83-1,00
Sexo										
Feminino	41.225	5.052.137	70,7	69,5-71,9	1,00	-	39,8	38,8-40,9	1,00	-
Masculino	33.364	5.095.563	38,0	36,7-39,4	0,53	0,51-0,56	13,4	12,4-14,4	0,32	0,29-0,36
Idade (anos)										
12-13	20.571	3.562.176	52,0	50,6-53,4	1	-	21,0	19,8-22,2	1,00	-
14-15	27.889	3.550.879	54,2	52,9-55,6	1,06	1,02-1,11	26,6	25,3-27,8	1,35	1,24-1,46
16-17	26.129	3.034.645	57,1	55,5-58,6	1,11	1,06-1,16	33,1	31,7-34,4	1,66	1,54-1,80
Cor da pele										
Branca	26.477	4.099.110	55,4	54,1-56,6	1,00	-	26,3	25,1-27,6	1,00	-
Preta	5.654	821.258	47,9	44,5-51,3	0,87	0,80-0,95	25,8	22,8-29,0	0,94	0,80-1,11
Parda	37.984	4.943.938	54,6	53,5-55,7	0,96	0,93-0,99	26,6	25,5-27,7	0,88	0,82-0,95
Amarela	1.881	215.840	54,0	49,1-58,8	0,99	0,90-1,09	26,4	22,9-30,3	0,96	0,83-1,11
Indígena	561	67.554	39,4	29,8-50,0	0,68	0,56-0,82	15,1	11,0-20,2	0,63	0,44-0,90
Classe econômica										
A (alta)	6.336	1.100.099	42,2	38,5-46,1	1,00	-	15,9	13,4-18,7	1,00	-
B	25.258	5.495.819	51,4	50,0-52,8	1,14	1,04-1,25	23,4	22,3-24,5	1,30	1,09-1,54
C	17.339	3.389.885	58,5	57,0-60,1	1,24	1,13-1,36	30,2	28,9-32,5	1,49	1,25-1,78
D-E (baixa)	936	161.898	64,6	58,0-70,6	1,33	1,17-1,51	32,7	26,8-39,2	1,49	1,15-1,93

ERICA: Estudo de Riscos Cardiovasculares em Adolescentes; RP: Razão de prevalência

^a^ Valores brutos.

^b^ População de adolescentes, segundo o Instituto Brasileiro de Geografia e Estatística.

^c^ Modelo ajustado por macrorregião, sexo, idade e classe econômica.

**Figura 1:** Prevalência (%) de inatividade física (< 300 min/semana) em adolescentes das 27 capitais brasileiras (barras verdes) e conjunto dos municípios com mais de 100 mil habitantes, não capitais das regiões, por sexo. ERICA, 2013-2014.

Entre os adolescentes brasileiros, 26,5% (IC95% 25,8-27,3) reportaram não realizar atividade física no lazer (zero min/semana), com prevalência maior no sexo feminino (39,8%, IC95% 38,8-40,9%) que no masculino (13,4%, IC95% 12,4-14,4) (Tabela 1). Em João Pessoa, capital com maior prevalência de nenhuma atividade física de lazer, um a cada três adolescentes não pratica qualquer atividade física no lazer, proporção que é de um para cada dois no sexo feminino (Figura 2).

**Figura 2:** Prevalência de nenhuma atividade física (zero min/semana) em adolescentes das 27 capitais brasileiras (barras verdes) e conjunto dos municípios com mais de 100 mil habitantes, não capitais das regiões, por sexo. ERICA, 2013-2014.

A Tabela 1 apresenta as prevalências e razões de prevalência ajustadas, no total da amostra, para inatividade física e nenhuma atividade física de acordo com as variáveis independentes. As maiores prevalências de inatividade física no lazer foram observadas na região Nordeste para nenhuma atividade física e nas regiões Nordeste e Sul quando o ponto de corte foi de pelo menos 300 min/semana. No modelo ajustado, a prevalência de inatividade física em ambas as categorias está associada de forma direta com a idade e de forma inversa com nível econômico (valor de p para tendência linear < 0,05).

Após estratificação por sexo, as maiores prevalências de inatividade física estiveram nas regiões Nordeste e Sudeste no sexo feminino e na região Sul entre os adolescentes do sexo masculino. Entre as meninas, quanto maior a idade (p < 0,001) e mais baixa a classe socioeconômica (p < 0,001), maior a prevalência de inatividade física. No sexo masculino, a prevalência de inatividade física tendeu a aumentar à medida que decrescia a condição socioeconômica (p = 0,003); autodeclarar-se como indígena, de cor da pele preta ou parda foram fatores de proteção à inatividade física no modelo ajustado (p < 0,05) (Tabela 2).

**Tabela 2:** Prevalências (%) e razões de prevalência para inatividade física (< 300 min/sem) no lazer por sexo, segundo macrorregião e características sociodemográficas em adolescentes. ERICA, Brasil, 2013-2014.

Variável	Inatividade física (< 300 min/sem)							

	Feminino				Masculino			
	
	%	IC95%	RP_ajustada_^a^	IC95%	%	IC95%	RP_ajustada_^b^	IC95%
Macrorregião								
Norte	65,4	63,7-67,1	1,00	-	34,1	32,5-35,8	1,00	-
Nordeste	72,0	69,9-74,0	1,13	1,08-1,19	39,3	35,8-42,9	1,15	1,01-1,30
Sudeste	72,0	69,8-74,1	1,16	1,11-1,22	37,8	35,8-39,7	1,15	1,05-1,26
Sul	69,7	67,8-71,6	1,12	1,06-1,18	42,1	38,3-46,0	1,19	1,05-1,34
Centro-Oeste	66,1	63,8-68,2	1,05	0,99-1,10	34,1	31,1-37,1	1,02	0,92-1,13
Idade (anos)								
12-13	66,3	64,5-68,1	1,00	-	38,0	35,4-40,6	1,00	-
14-15	72,5	70,7-74,3	1,10	1,06-1,15	36,1	34,3-38,0	0,99	0,90-1,10
16-17	73,8	71,9-75,6	1,12	1,07-1,18	40,3	38,2-42,4	1,09	0,99-1,20
Cor da pele								
Branca	70,9	69,2-72,5	1,00	-	40,4	38,3-42,6	1,00	-
Preta	67,4	63,1-71,4	0,96	0,88-1,03	34,4	30,8-38,2	0,75	0,64-0,89
Parda	71,3	69,9-72,6	0,99	0,96-1,03	36,5	34,8-38,2	0,91	0,84-0,98
Amarela	65,5	58,9-71,5	0,94	0,86-1,02	41,7	34,7-49,0	1,08	0,89-1,30
Indígena	62,0	51,4-71,5	0,86	0,71-1,05	29,3	16,4-46,4	0,52	0,38-0,71
Classe econômica								
A (alta)	59,4	55,1-63,6	1,00	-	31,4	27,0-36,2	1,00	-
B	68,1	66,4-69,8	1,13	1,03-1,23	35,8	33,9-37,8	1,17	1,00-1,37
C	73,5	71,4-75,4	1,22	1,13-1,32	39,9	37,3-42,6	1,33	1,12-1,59
D-E (baixa)	80,0	73,9-85,0	1,33	1,20-1,48	40,5	29,0-53,1	1,34	0,96-1,87

ERICA: Estudo de Riscos Cardiovasculares em Adolescentes; RP: Razão de prevalência

^a^ Modelo ajustado por macrorregião, idade e condição econômica.

^b^ Modelo ajustado por macrorregião, idade, condição econômica e cor da pele.

Em relação à prática de nenhuma atividade física (Tabela 3), também observou-se um gradiente crescente da prevalência de nenhuma atividade física no lazer com o aumento da idade, em ambos os sexos (p < 0,001). Pior condição socioeconômica associou-se a maiores prevalências de nenhuma atividade física no lazer apenas entre as adolescentes do sexo feminino (p = 0,001).

**Tabela 3:** Prevalências (%) e razões de prevalência para nenhuma atividade física (0 min/sem) no lazer por sexo, segundo macrorregião e características sociodemográficas em adolescentes. ERICA, Brasil 2013-2014.

Variável	Nenhuma atividade física (0 min/sem)							

	Feminino				Masculino			
	
	%	IC95%	RP_ajustada_^a^	IC95%	%	IC95%	RP_ajustada_^b^	IC95%
Macrorregião								
Norte	39,8	38,3-41,3	1,00	-	14,6	13,3-16,1	1,00	-
Nordeste	44,0	42,1-46,0	1,17	1,08-1,27	15,0	13,1-17,2	0,99	0,80-1,23
Sudeste	39,9	38,2-41,7	1,07	0,98-1,17	13,2	11,5-15,0	0,90	0,72-1,13
Sul	34,6	31,1-38,2	0,90	0,78-1,06	11,5	10,3-12,7	0,74	0,58-0,93
Centro-Oeste	35,6	33,2-38,2	0,96	0,87-1,06	11,5	10,1-13,1	0,77	0,62-0,95
Idade (anos)								
12-13	31,5	29,6-33,5	1,00	-	10,6	9,3-13,4	1,00	-
14-15	41,1	39,2-43,1	1,32	1,19-1,45	12,2	10,8-13,7	1,47	1,20-1,80
16-17	48,0	46,3-49,6	1,56	1,42-1,72	18,0	16,4-19,8	2,01	1,68-2,41
Cor da pele								
Branca	39,3	37,3-41,4	1,00	-	13,8	12,5-15,3	1,00	-
Preta	42,5	38,4-46,7	0,97	0,84-1,13	14,2	10,9-18,4	0,87	0,59-1,27
Parda	39,7	38,1-41,3	0,91	0,84-0,98	12,4	11,2-13,8	0,80	0,69-0,93
Amarela	38,4	32,9-44,1	0,96	0,83-1,11	13,7	9,8-18,8	0,96	0,63-1,48
Indígena	36,0	26,9-46,3	0,80	0,55-1,17	5,5	3,2-9,3	0,37	0,19-0,73
Classe econômica								
A (alta)	25,9	21,2-31,3	1,00	-	9,5	7,2-12,5	1,00	-
B	35,6	33,7-37,5	1,34	1,09-1,65	12,0	10,7-13,5	1,29	0,97-1,70
C	44,2	42,0-46,4	1,60	1,30-1,97	12,7	11,0-14,5	1,32	0,95-1,83
D-E (baixa)	43,4	34,5-52,7	1,54	1,18-2,00	16,1	10,4-24,2	1,51	0,88-2,59

ERICA: Estudo de Riscos Cardiovasculares em Adolescentes; RP: Razão de prevalência

^a^ Modelo ajustado por macrorregião, idade e condição econômica.

^b^ Modelo ajustado por macrorregião, idade, cor da pele e condição econômica.

## DISCUSSÃO

Os dados do ERICA mostram que mais da metade dos adolescentes brasileiros moradores de cidades de médio e grande porte não atinge a recomendação de pelo menos 300 min/semana de atividade física para promoção da saúde. Esse percentual é ainda maior entre as meninas, ultrapassando 70,0%. Igualmente preocupante é o fato de um a cada quatro adolescentes não praticarem atividade física no lazer, prevalência que se aproxima de 50,0% entre as meninas de algumas capitais.

A mensuração dos níveis populacionais de atividade física, especialmente na infância e adolescência, é bastante complexa e a comparação entre os estudos é difícil. O uso de questionários nem sempre produz estimativas precisas, o que pode ocasionar erros de classificação, e sua concordância com medidas diretas é apenas parcial^21^. No entanto, a utilização de métodos de mensuração direta, como acelerometria, seria inviável num estudo como o ERICA devido ao alto custo e à complexidade logística envolvida. A utilização de um instrumento já validado^12^ aumenta a confiabilidade dos dados, no entanto isso não reduz a dificuldade de comparações devido aos diferentes instrumentos utilizados e os diferentes domínios da atividade física avaliados em cada estudo. Neste estudo, optou-se por investigar apenas a atividade física no lazer, pois é o domínio mais explorado no questionário do ERICA e, se comparado aos demais, é o domínio mais importante para o cumprimento das recomendações de atividade física entre adolescentes brasileiros^19^.

A literatura científica mostra que a prática de atividade física na adolescência está associada a inúmeros benefícios à saúde durante a adolescência e com reflexos igualmente importantes na idade adulta^11^ ^,^ ^15^ ^,^ ^18^. Apesar de muitos estudos utilizarem a recomendação de pelo menos 300 min/semana em atividade física moderada a vigorosa para promoção da saúde em adolescentes, evidências suportam que volumes menores já podem trazer benefícios. Essas variações são sustentadas pela origem multicausal de cada morbidade, sobretudo nas doenças crônicas, bem como pelos diferentes mecanismos fisiológicos que podem relacionar a atividade física com determinada morbidade. Revisão sistemática mostrou que 30 min/dia de atividade física são suficientes para obtenção de benefícios à saúde cardiovascular entre adolescentes^16^. Entretanto, ensaio clínico randomizado envolvendo jovens com excesso de peso e inativos, apontou que 20 min de atividade física aeróbica, praticadas cinco vezes na semana, durante 13 semanas, já reduz o risco de desenvolver diabetes, o percentual geral de gordura e a gordura visceral, bem como melhora a aptidão física, se comparado ao grupo controle que mantinha a rotina habitual^8^.

Intervenções que estimulem a transição da inatividade física total para um estágio de ação, independentemente do volume de atividade física inicialmente praticado, podem promover impactos imediatos. Essa estratégia pode funcionar de maneira complementar a programas que visam à manutenção e ao aumento gradual da prática de atividade física entre adolescentes. O domínio do lazer é propício para o desenvolvimento de intervenções na adolescência.

Nesse contexto, a escola parece ser o espaço ideal para condução dessas intervenções, devido à grande concentração de adolescentes, segurança, existência de espaço físico e profissionais capazes de estimular e supervisionar essas atividades. Aumentar o tempo de prática efetiva durante as aulas de educação física e torná-las mais atrativas deve ser uma meta, bem como reduzir o número de dispensas das aulas. Outras ações como interrupções do tempo sedentário durante as aulas, proposição de intervalos ativos e atividades extracurriculares são estratégias que têm sido estudadas e poderiam aumentar a prática de atividades físicas nas escolas e ter reflexos importantes fora delas^6^. No entanto, essas ações precisam estar conectadas a um aumento do suporte social para prática e melhoria do acesso, tornando-o socialmente igualitário, especialmente em países de condições socioeconômicas tão heterogêneas como o Brasil.

O interesse pelo estudo da epidemiologia da (in)atividade física no Brasil é crescente, com a maioria das publicações concentradas no eixo Sul-Sudeste e recente crescimento das publicações na região Nordeste. Entretanto, ainda são raros os estudos nessa temática conduzidos nas regiões Norte e Centro-Oeste do País^2^. No ERICA, essas regiões apresentaram as menores prevalências de inatividade física no lazer, independente do sexo. A última edição da PeNSE também observou maior prática de atividades de lazer na região Norte e relacionou esse resultado a questões culturais e características urbanas favoráveis nem sempre encontradas em regiões mais urbanizadas como Sudeste, Nordeste e Sul do País^19^. No entanto, são necessários mais estudos nessas regiões para um melhor entendimento dos fatores associados e promotores da prática de atividade física.

Maior prevalência de inatividade física no sexo feminino é frequentemente observada, especialmente no lazer^3^ ^,^ ^14^ ^,^ ^19^. O mesmo ocorre com o aumento da idade^10^, sendo ainda mais pronunciada na transição da adolescência para a idade adulta^1^ ^,^ fase que os adolescentes estudados ainda irão vivenciar. O nível socioeconômico esteve associado à inatividade física entre as meninas, principalmente em relação a não atingir a recomendação de pelo menos 300 min/semana, reforçando as interpretações que relacionam essa variável à oportunidade de praticar atividade física estruturada (compra de equipamentos, transporte, pagamento de mensalidades, entre outros) e suporte emocional e social da família (como permissão, estímulo, companhia para prática e maior facilidade de acesso à informação)^9^.

Os primeiros dados sobre inatividade física do ERICA ajudam a dimensionar quão desafiador é esse problema no Brasil. Esses resultados são úteis para o direcionamento de ações que busquem reduzir a inatividade física em adolescentes brasileiros. Futuras análises do ERICA poderão expandir esse conhecimento avaliando, e.g., a importância da estrutura escolar, sazonalidade e variações climáticas, e a relação da inatividade física com diversos fatores de risco cardiovascular pesquisados.

O investimento em políticas de estímulo à atividade física em adolescentes deve ser tratado como prioridade nas agendas governamentais relativas ao esporte, à saúde e à educação, bem como de órgãos de fomento à pesquisa. Ações governamentais como os programas Segundo Tempo, Mais Educação e Atleta na Escola são passos importantes e que devem ser expandidos. A construção do projeto de Lei de Diretrizes e Bases do Sistema Nacional do Esporte, que visa tornar o investimento no esporte em política governamental a fim de massificar a prática esportiva no País, pode ser igualmente favorável.

Esses recursos devem auxiliar na mudança do cenário atual de forma abrangente, tendo na escola um parceiro importante para o fomento de atividades que incluam toda a comunidade escolar. O desenvolvimento de ações que visem à transição daqueles que não praticam nenhuma atividade física para adoção de alguma prática deve ser prioridade, mesmo que essas pessoas não venham a atingir, em um primeiro momento, as recomendações de atividade física para saúde.
